# Flower‐visitor communities of an arcto‐alpine plant—Global patterns in species richness, phylogenetic diversity and ecological functioning

**DOI:** 10.1111/mec.14932

**Published:** 2018-12-07

**Authors:** Mikko Tiusanen, Tea Huotari, Paul D. N. Hebert, Tommi Andersson, Ashley Asmus, Joël Bêty, Emma Davis, Jennifer Gale, Bess Hardwick, David Hik, Christian Körner, Richard B. Lanctot, Maarten J. J. E. Loonen, Rauni Partanen, Karissa Reischke, Sarah T. Saalfeld, Fanny Senez‐Gagnon, Paul A. Smith, Ján Šulavík, Ilkka Syvänperä, Christine Urbanowicz, Sian Williams, Paul Woodard, Yulia Zaika, Tomas Roslin

**Affiliations:** ^1^ Department of Agricultural Sciences University of Helsinki Helsinki Finland; ^2^ Centre for Biodiversity Genomics Biodiversity Institute of Ontario University of Guelph Guelph Ontario Canada; ^3^ Kevo Subarctic Research Station Biodiversity Unit University of Turku Turku Finland; ^4^ Department of Ecology, Evolution and Behavior University of Minnesota Minneapolis Minnesota; ^5^ Department of Biology University of Texas at Arlington Arlington Texas; ^6^ Département de Biologie and Centre d’études Nordiques Université du Québec à Rimouski Rimouski Quebec Canada; ^7^ Department of Geography University of Guelph Guelph Ontario Canada; ^8^ East Bay Southampton Island Shorebird Crew National Wildlife Research Center Environment Canada Ottawa Ontario Canada; ^9^ Department of Biosciences University of Helsinki Helsinki Finland; ^10^ Department of Biological Sciences Simon Fraser University Burnaby British Columbia Canada; ^11^ Department of Environmental Sciences Institute of Botany University of Basel Basel Germany; ^12^ U.S. Fish and Wildlife Service Anchorage Alaska; ^13^ Arctic Centre University of Groningen Groningen The Netherlands; ^14^ Kilpisjärvi Biological Station University of Helsinki Kilpisjärvi Finland; ^15^ Conservation Ontario Newmarket Ontario Canada; ^16^ Département des Sciences du Bois et de la Forêt Université Laval Quebec City Québec Canada; ^17^ Wildlife Research Division, Environment and Climate Change Canada Ottawa Ontario Canada; ^18^ Department of Environmental Sciences Faculty of Engineering and Science Western Norway University of Applied Sciences Sogndal Norway; ^19^ Natural History Museum University of Oslo Oslo Norway; ^20^ Department of Biology Dartmouth College Hanover New Hampshire; ^21^ Kluane Lake Research Station Yukon Silver City, Yukon Canada; ^22^ Canadian Wildlife Service, Environment and Climate Change Canada/Government of Canada Yellowknife Northwest Territories Canada; ^23^ Department of Geography Khibiny Academic Research Station Lomonosov Moscow State University Moscow Russia; ^24^ Department of Ecology Swedish University of Agricultural Sciences Uppsala Sweden

**Keywords:** arctic ecology, DNA barcoding, *Dryas*, ecosystem functioning, flower visitor, pollination

## Abstract

Pollination is an ecosystem function of global importance. Yet, who visits the flower of specific plants, how the composition of these visitors varies in space and time and how such variation translates into pollination services are hard to establish. The use of DNA barcodes allows us to address ecological patterns involving thousands of taxa that are difficult to identify. To clarify the regional variation in the visitor community of a widespread flower resource, we compared the composition of the arthropod community visiting species in the genus *Dryas* (mountain avens, family Rosaceae), throughout Arctic and high‐alpine areas. At each of 15 sites, we sampled *Dryas* visitors with 100 sticky flower mimics and identified specimens to Barcode Index Numbers (BINs) using a partial sequence of the mitochondrial COI gene. As a measure of ecosystem functioning, we quantified variation in the seed set of *Dryas*. To test for an association between phylogenetic and functional diversity, we characterized the structure of local visitor communities with both taxonomic and phylogenetic descriptors. In total, we detected 1,360 different BINs, dominated by Diptera and Hymenoptera. The richness of visitors at each site appeared to be driven by local temperature and precipitation. Phylogeographic structure seemed reflective of geological history and mirrored trans‐Arctic patterns detected in plants. Seed set success varied widely among sites, with little variation attributable to pollinator species richness. This pattern suggests idiosyncratic associations, with function dominated by few and potentially different taxa at each site. Taken together, our findings illustrate the role of post‐glacial history in the assembly of flower‐visitor communities in the Arctic and offer insights for understanding how diversity translates into ecosystem functioning.

## 1 INTRODUCTION

How community structure translates into ecosystem functioning is an essential and topical question (Brose & Hillebrand, 2016; Cardinale et al., 2006, 2012; Duncan, Thompson, & Pettorelli, 2015; Oliver et al., 2015; Tilman, Isbell, & Cowles, 2014; Wang & Brose, 2018). A general positive link between biodiversity and ecosystem functioning (Allan et al., 2015; Butterfield, Camhi, Rubin, & Schwalm, 2016) has been variously ascribed to the effects of diversity per se (e.g., more species complement each other's use of available resources, thus allowing more complete resource use; e.g., Tilman et al., 2001) versus effects of species identity (with some species being particularly efficient from a functional perspective; e.g., Cardinale, Palmer, & Collins, 2002; Hooper, Chapin, & Ewel, 2005). Overall, species within a community often have varying impacts on individual ecosystem functions (Cardinale et al., 2006; Piccini et al., 2018; Slade et al., 2017).

In general, biodiversity decreases with an increase in latitude (MacArthur, 1972; Pianka, 1966). As a result, communities in the Arctic are less diverse than those at lower latitudes. Yet, several additional processes contribute to shape patterns of diversity. Local communities assemble as a function of both stochastic and deterministic processes (Götzenberger et al., 2012; Gravel, Canham, Beaudet, & Messier, 2006; Leibold & McPeek, 2006; Weiher et al., 2011). These assemblies are a result of neutral processes, historical processes such as speciation, species dispersal, abiotic environmental factors and biotic interactions (Götzenberger et al., 2012; Weiher et al., 2011). As an outcome of these assembly processes, the number, abundance, identities and traits of the species present in local communities vary in space and/or time.

When considering community assembly processes in the Arctic, it is important to consider that climatic conditions have been and still are in constant change. During the Pleistocene, fluctuating ice cover affected both the environment and the species present (Abbott et al., 2000; Hultén, 1937). During glacial maxima, the distributions of many species retreated to ice‐free refugia (Hopkins, 1967), whereas in interglacial periods, species moved northwards (Frenzel, 1968; Hultén, 1937). Thus, the Arctic fauna is currently recovering from the last glaciation period, and the modern fauna at a given site is potentially affected by the distance from past glacial refugia.

Understanding the drivers of local community structure and the role of this structure for ecosystem functioning helps us to predict how communities and their functioning react to ongoing species loss and environmental change (Kattsov et al., 2005; Memmott, Craze, Waser, & Price, 2007; Post et al., 2009). While recent studies have focused on animal diversity–functioning relationships (e.g., Orford, Murray, Vaughan, & Memmott, 2016; Wang & Brose, 2018; Winfree et al., 2018), there remain critical knowledge gaps in understanding the links between biodiversity and ecosystem functioning in natural, large‐scale and unmanipulated systems (Winfree et al., 2018). Pollination is an ecosystem function of global importance. Therefore, studying natural flower‐visitor communities across the species‐poor Arctic provides an intriguing large‐scale study system for understanding how flower‐visitor diversity impacts ecosystem functioning.

**Figure 1:**
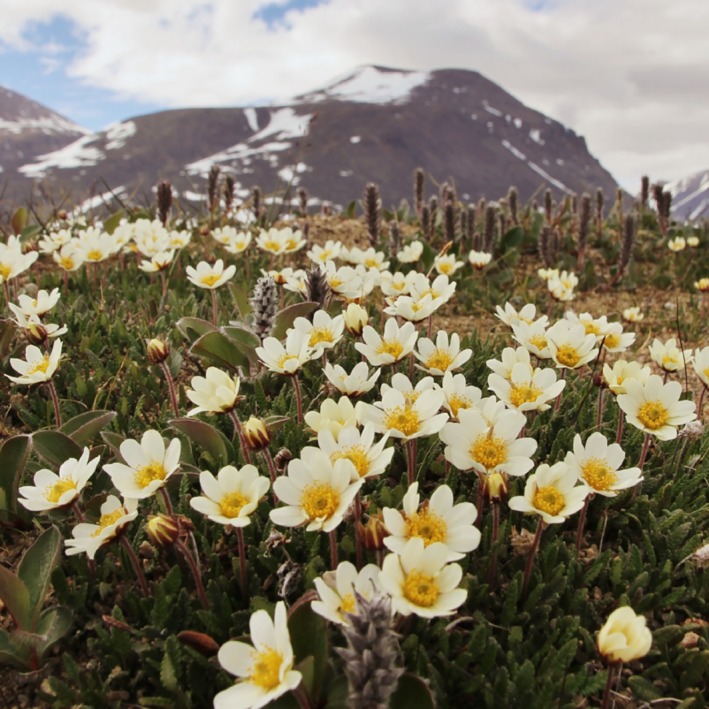
*D. integrifolia x octopetala* growing in Zackenberg, NE Greenland

In this paper, we examine the key drivers behind regional variation in the flower‐visitor community of Mountain Avens, *Dryas* spp. (Rosaceae, Figure 1), an important flower resource in cold regions of the Northern hemisphere (Lundgren & Olesen, 2005; Olesen, Bascompte, Elberling, & Jordano, 2008; Rasmussen, Dupont, Mosbacher, Trøjelsgaard, & Olesen, 2013). Using state‐of‐the‐art molecular tools to describe the taxonomic and phylogenetic composition of communities, we compare the structure and functioning of the arthropod community visiting *Dryas* at 15 sites distributed across arctic and alpine areas. We specifically ask:

1. Who visits these abundant arctic flowers? Our previous work at a single arctic site has identified *Dryas* as a key resource for a large fraction of the local arthropod fauna (Schmidt et al., 2017; Tiusanen, Hebert, Schmidt, & Roslin, 2016). Thus, we expect *Dryas* at sites across the Arctic to be visited by a locally diverse arthropod fauna, but the exact composition of the local flower‐visiting community to vary with geographic variation in the local species pool.
2. How does the structure of the flower‐visitor community vary in space? Since the fauna of the Arctic is subject to harsh climates, we expect a general latitudinal gradient in species richness, with strong imprints of two climatic descriptors in particular: temperature (as a general metric of energy available to ectotherms in general) and precipitation (e.g., Gaston, 2000; Hillebrand, 2004). With respect to the latter, we note that much of arctic productivity is strongly limited by water availability, with extensive areas of desert or semidesert dominating large parts of the Arctic (Jonasson, Callaghan, Shaver, & Nielsen, 2000).
3. How are these flower‐visitor communities assembled? A priori, we predict that the same geological processes and colonization routes which have shaped arctic plant communities (cf. Alsos et al., 2015; Eidesen et al., 2013) have also moulded the insect communities associated with them. Thus, we expect a strong correlation in patterns of pairwise floristic and faunistic similarity among arctic sites.
4. Is community structure reflected in function (i.e., pollination)? Based on general biodiversity vs ecosystem functioning (BEF) relations (Hooper et al., 2005; Loreau et al., 2001; Tilman et al., 2014), we expected ecosystem functioning (sensu seed set by *Dryas*) to increase with an increase in the diversity of flower‐visitors. In particular, we expected phylogenetically more diverse communities to convey improved functioning, as being composed of functionally more complementary taxa. We expect such communities to provide stronger facilitation among taxa (Tilman et al., 1997, 2001) and stronger functional redundancy, that is, more stable ecosystem functioning (Brittain, Kremen, & Klein, 2013; Evans, Pocock, & Memmott, 2013; Oliver et al., 2015) in the rapidly variable (Kankaanpää et al., 2018) arctic climate.

## 2 MATERIALS AND METHODS

### 2.1 Target plant

As a model taxon of arctic pollination, we selected Mountain Avens, *Dryas* spp (including *D. drummondi*,* D. integrifolia*,* D. octopetala* and hybrids of the latter two taxa; see Table 1). Plants in this genus are perennial dwarf shrubs, abundant in many arctic and alpine areas (Philipp & Siegismund, 2003). *Dryas* flowering starts shortly after snowmelt, with most individuals having flowered within a month.

**Table 1:** Study sites included in the sampling of flower visitors of *Dryas* and the quantification of *Dryas* seed set success. The first column identifies the research station or other site where the sampling was conducted. Columns “Longitude” and “Latitude” report the location of the specific study site in WGS‐84 coordinates; column “Elevation” provides the altitude of the study site as metres above the sea level; column “Flower visitors” gives the number of individuals caught on the *Dryas* flowers deployed at each site; and column “Species” gives the number of flower‐visiting taxa identified using Barcode Index Numbers (BINs). The two columns, “Seed set P” and “Seed Set E,” identify the *Dryas* seed set data collected in the presence of pollinators (P) and in pollinator exclosures (E), respectively, with the slash separating the number of successful and the total number of flowers inspected after subtracting all male‐only flowers, respectively. The last column “*Dryas* species” identifies the species of *Dryas* occurring at each study site. Data collection was conducted between June and August in 2014 at all study sites. See Figure 2 for a map of locations. Here, “*D. integrifolia & D. octopetala”* indicates that both species occur at the site, “*D. integrifolia x octopetala*” that most individuals are, in fact, hybrids between the two

Research station^a^	Country	Longitude	Latitude	Elevation	Flower visitors	Species	Seed Set P	Seed Set E	*Dryas* species
Bylot Island	Canada	73.15	−79.98	20	651	82	515/708	75/115	*D. integrifolia*
Coats Island	Canada	62.85	−82.48	39	1389	160	NA	NA	*D. integrifolia*
East Bay	Canada	63.99	−81.69	2	587	93	349/529	25/55	*D. integrifolia*
Finse	Norway	60.61	7.53	1435	6636	99	297/382	NA	*D. octopetala*
Furka	Switzerland	46.58	8.42	2480	1211	164	90/225	3/46	*D. octopetala*
Kangerlussuaq^b^	Greenland	67.13	−50.16	328	136	14	797/880	NA	*D. integrifolia*
Kevo	Finland	69.94	26.54	362	1518	141	56/212	10/41	*D. octopetala*
Khibiny	Russia	67.59	33.7	960	2469	255	NA	NA	*D. octopetala*
Kilpisjärvi	Finland	69.08	20.81	797	2944	226	78/192	24/63	*D. octopetala*
Kluane Lake	USA	61.02	−138.34	876	1419	124	241/335	13/31	*D. drummondi*
MacKenzie Delta	Canada	69.37	−134.88	12	9974	133	NA	NA	*D. integrifolia*
Ny‐Ålesund	Svalbard	78.93	11.9	12	948	50	69/197	24/65	*D. octopetala*
Toolik Lake	USA	68.37	−149.32	835	367	102	149/325	5/46	*D. integrifolia & D. octopetala*
Utqiaģvik (Barrow)	USA	71.26	−156.56	3	775	83	144/227	0/145	*D. integrifolia & D. octopetala*
Zackenberg	Greenland	74.51	−20.53	213	321	46	219/735	6/97	*D. integrifolia x octopetala*

a The full name and description of each station is found in the INTERACT station catalogue (https://eu-interact.org/publication/test-publication/).

b Due to poor weather conditions, this site was excluded from all phylogenetic analyses.

*Dryas* are monoicous plants and are known to exhibit varying levels of autogamy. A limited fraction of local flowers will be unisexual (i.e., male‐only or female‐only), but the male‐only flowers were explicitly excluded from the current study (see section *Success of seed set*). Access to flower visitors generally increases seed set (by either outcrossing or an increased level of autogamy (Hocking & Sharplin, 1965; Kevan, 1972; Lundemo & Totland, 2007; Tiusanen et al., 2016); but see also (Wada, 1999), where environmental factors emerged as the driver of seed set in one location).

In terms of their morphology, flowers of *Dryas* are morphologically simple, large and open, consistently coloured in white and yellow, and provide easy access for many types of pollinators (Figure 1). As a result, *Dryas* have proven key taxa of pollination networks in many regions of the Arctic (Lundgren & Olesen, 2005; Olesen et al., 2008; Rasmussen et al., 2013; Tiusanen et al., 2016). Given the generally low species richness of the arctic ecosystems, it then seems reasonable to hypothesize that higher diversity in the flower‐visiting community would ensure the presence at least *some* particularly efficient pollinator species in the community and/or the presence of mutually complementary species, which together ensure higher average functioning. By targeting genus *Dryas* as our model system, we may thus address essential questions on arctic flower‐visitor communities across large spatial scales.

### 2.2 Sampling sites

**Figure 2:**
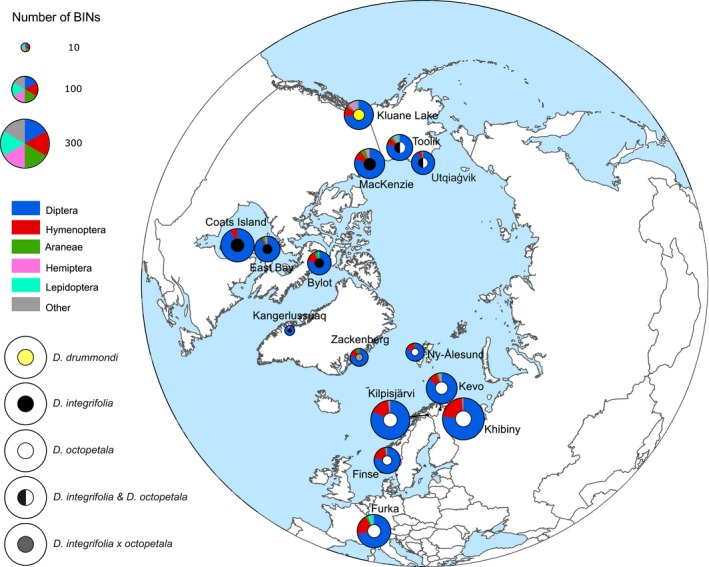
The location of sampling sites across arctic and alpine areas, with order‐level composition of local flower‐visitor communities shown by pie charts. The size of each circle represents the number of species caught, whereas sectors indicate the relative proportion of each taxon. The total number of individuals caught was 31,345, and the total number of species was 1,360. The identity of the *Dryas* taxa examined at the respective sites (Table 1) is shown by the colour of the centre of the pie charts (see legend in figure)

To resolve large‐scale patterns in the arthropod communities visiting flowers of *Dryas*, we drew on a large‐scale collaboration among 15 research stations belonging to the INTERACT network (https://eu-interact.org/). The sampling locations extended in elevation from 0 to 2,480 m above sea level (Table 1, Figure 2). To quantify small‐ and large‐scale variation in seed set and in the flower‐visitor community, we placed five 1 m × 1 m study plots within each of the 15 study locations (i.e., 5 × 15 = 75 study squares in total; for study square, see Figure S1, Supplemental information). The study plots were distributed at least 1–2 m apart from each other. All sampling instructions were distributed through and are archived on a webpage (http://www.helsinki.fi/foodwebs/dryas/index.htm, Hardwick, Tiusanen, & Roslin, 2013; Appendix S1, Supplemental information).

### 2.3 Sampling of flower visitors

To establish how the composition of the flower‐visitor communities varies in space, we sampled visitors using sticky mimics of *Dryas* flowers (see Tiusanen et al., 2016; Figure S2, Supplemental information). This trap design was originally tested in Greenland 2013 by Visakorpi et al. (2014), who compiled a large material of 2,825 trap hours with different versions of sticky traps and 125 hr of observations of real flowers or real flowers sprayed with glue. Among these sets, we found no detectable differences in visitation rates to trap flowers and real *Dryas* flowers. In terms of flies alone, trap flowers caught 0.11 ± *SE* 0.11 individuals per hour as compared to 0.05 ± *SE* 0.08 flies per hour observed on real flowers. In terms of all flower‐visiting taxa, trap flowers caught 0.12 ± *SE* 0.12 arthropods per hour whereas real *Dryas* flowers were visited by 0.17 ± *SE* 21 arthropods per hour (see Visakorpi et al., 2014). Neither visitation rates nor the species composition of samples (see Table S1, Figure S3, Supplemental information) differed detectably between methods. By thus adequately sampling the local flower‐visitor community, this method provides multiple advantages of the traditional method of visually observing insect visits to focal flowers (e.g., Ballantyne, Baldock, & Willmer, 2015; Cirtwill, Roslin, Rasmussen, Olesen, & Stouffer, 2018; Rasmussen et al., 2013; Vázquez, Morris, & Jordano, 2005): by using the sticky flower mimics, we were able to acquire larger samples, sample many regions at the same time and identify all the trapped visitors to the species level through tissue sampling and DNA barcoding.

Each of the traps was made of two circular pieces of sticky paper: a white piece (ø30 mm; made of Sticky Roll, Barrettine Environmental Health, Bristol, UK) to represent the petals and a yellow piece (ø8 mm; Yellow Sticky Board, Barrettine Environmental Health, Bristol, UK) to represent the stamen. We equipped each flower with a short stem made out of iron wire and stuck it into the soil so it was at the level of natural flowers. Within each of the 15 study sites, 20 such mimics were placed in *Dryas* tussocks among the real flowers in each of the five study squares (5 × 20 = 100 mimics/study site; for illustrations, see Figure S2, Supplemental information). Sampling of flower visitors was conducted during peak flowering of *Dryas*. The flowering peak at each location was scored when half of the flower heads were open. The traps were kept in the field for three days (72 hr).

### 2.4 Identification of flower visitors

We used DNA barcoding to resolve the species diversity and to characterize the phylogenetic composition of flower‐visitor communities. To do this, we first sequenced the standard barcode region (Hebert, Cywinska, Ball, & DeWaard, 2003) of the mitochondrial cytochrome *c* oxidase 1 (COI) gene region of the flower visitors and then compared these sequences to a reference library in BOLD (The Barcode of Life Data Systems, www.barcodinglife.org, Ratnasingham & Hebert, 2007). For the DNA barcode analysis, DNA extraction, amplification and sequencing were implemented in accordance with the standard protocols at the Canadian Center for DNA Barcoding (CCDB; Appendix S2, Supplemental information). Taxa were identified using the ID engine of BOLD using Barcode Index Numbers (BINs) as taxonomical units (Ratnasingham & Hebert, 2013). With some exceptions, a BIN equals a morphologically identifiable species (Wirta et al., 2016), and for simplicity, we henceforth refer to them as “species.” If more than 10 morphologically identical specimens were encountered at a trap, we subsampled a proportion of them and multiplied the identification result by the number of individuals of the traps subsampled at the corresponding site. The flower visitors sampled were individually labelled and stored at the Department of Agricultural Sciences in the University of Helsinki. Because any finite sample is unlikely to recover all arthropod species present at a site, we derived Chao 1 (Chao, 1984, 1987) estimates of asymptotic species richness using function Chao 1 in package *fossil* (Vavrek, 2012) in r (The R Core Team, 2016).

### 2.5 Drivers of flower‐visiting species richness across the Arctic

To identify the environmental factors likely affecting species richness of flower visitors in an area, we extracted environmental variables for each site from the WorldClim database (version 2.0, Fick & Hijmans, 2017). The 19 variables extracted characterized two broad groups of abiotic conditions: temperature (11 variables) and precipitation (8 variables), with individual variables focusing on different parts of the year. To reduce the dimensionality of the data, we then used principal component analysis (PCA). To derive components with a clear relation to particular climatic axes, we formed separate principal components for variables related to temperature and precipitation, respectively. The two PCAs were derived in r, in each case using the variance–covariance method. Of the variance in temperature and precipitation, 53.1% and 85.1% were explained by the first principal components of the PCAs (Temperature PC 1, Precipitation PC 1), respectively. The resultant axes and individual factor loadings are shown in Tables S2 and S3 and in Figures S4 and S5 (Supplemental information). To identify the climatic drivers of species diversity across the Arctic, we then built a piecewise structural equation model (SEM, Lefcheck, 2016) of Chao 1 estimates (Chao, 1984, 1987) of species richness per study site. In the SEM, we fitted separate general linear models (GLM) with the Chao 1 value as a response variable and the first principal components of temperature and precipitation as explanatory variables. We also fitted GLMs where the latitude, altitude and their interaction were used to explain the first principal components of temperature and precipitation (see Figure S6, Supplemental information, for relationship of Temperature PC 1 and Precipitation PC 1). Since two alpine sites (Finse and Furka, Table 1) were characterized by environmental conditions strongly different from all other sites and might thus exert disproportionate leverage on any joint analyses, we excluded them from these analyses. By this approach, we explicitly avoided the potential for any spurious patterns caused by single data points. Kangerlussuaq was excluded from the analyses due to poor weather conditions during the sampling of flower visitors. The piecewise SEM was fitted with package *piecewiseSEM* (Lefcheck, 2016) in r.

### 2.6 Success of seed set

As a metric of ecosystem functioning, we recorded the per capita success of individual *Dryas* flowers at each study site in establishing seed heads (hereafter called seed set). By recording the seed set, we were able to get an overall picture of the pollination process (Ne'eman, Jürgens, Newstrom‐Lloyd, Potts, & Dafni, 2010). We counted all flower heads at the start of sampling, subtracting all male‐only flowers (i.e., flowers lacking pistils) from the total number of flowers to arrive at the number of flowers potentially available to produce seeds. At the end of the season, we counted all seed heads of *Dryas* in the study squares and classified them according to whether they had generated viable seeds or not (for illustrations of categories, see Figure S7, Supplemental information). Potential wind pollination and autogamy of *Dryas* were examined by recording seed set rate of plants kept under pollinator exclusion. For this purpose, we constructed small cylinder‐shaped mesh tents of light and neutrally coloured fabric (⌀ 20 cm, mesh size, 0.3 mm × 0.3 mm, Eurokangas, Marley T300; for illustrations, see Appendix S1 & Figure S1, Supplemental information). The lightweight fabric was selected not to affect the growth of the plants, stop the wind or attract flower visitors to nearby plants. Two insect‐excluding mesh tents were placed in each study square, while the buds were still closed. At the end of the season, the seed heads inside the tents were counted and divided into the same categories as outlined above (Figure S7, Supplemental information). As an estimate of the role of arthropods in *Dryas* pollination, we compared seed set success for flowers located inside and outside the pollinator exclusions. What little effect the tents’ fabric had on internal conditions was likely to decrease wind speed, which will in fact increase the temperature inside the exclusions. Overall, as warmer temperatures are related to increased seed set in *Dryas* (Wada, 1999; Welker & Molau, 1997), the exclusion cages used here may slightly increase the nonpollinator‐mediated component of the seed set inside the exclusion cages, biasing our estimates of the insect‐induced increase in seed set facilitation in a conservative rather than liberal direction. Seed set success was not recorded at Coats Island, Khibiny and MacKenzie, while pollinator exclusion was not used at Finse and Kangerlussuaq (Figure 5); all of these sites were thus excluded from further seed set analyses.

### 2.7 Consistency in faunistic vs floristic patterns across the Arctic

To compare flower‐visiting arthropods to patterns among vascular plants across the Arctic, we compiled lists of vascular plant species present at each of the research sites. To standardize the nomenclature, we used the Pan Arctic Flora Checklist (Elven, Murray, Razzhivin, & Yurtsev, 2011) and used species‐level classifications. For a full list of vascular plant species, see Table S5 (Supplemental information). To examine the consistency in faunistic (flower‐visitor species) and floristic (vascular plant species) patterns across the Arctic, we compared pairwise faunistic similarity (Jaccard index based on shared arthropod species) to pairwise floristic similarity (Jaccard index based on shared plant species) using Mantel test. These tests were implemented in package *vegan* (Oksanen et al., 2016) in r.

### 2.8 Phylogenetic data

**Figure 3:**
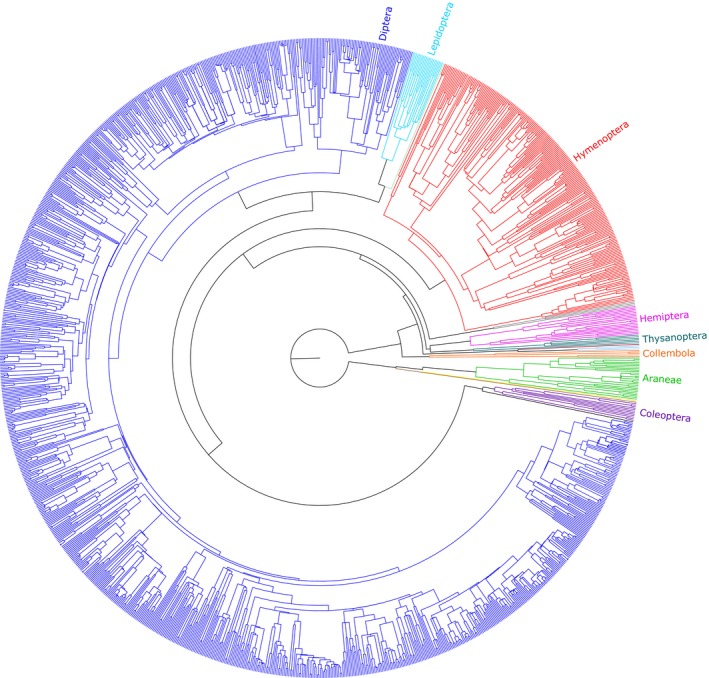
Bayesian phylogeny of arthropod species visiting *Dryas* flowers across the Arctic. Colours identify the most abundant orders detected in the sampling. For details on calibration of the phylogenetic tree, see Appendix S3, Supplemental information

To describe phylogenetic relations among all flower‐visitor taxa encountered, we constructed a Bayesian phylogeny (Figure 3) based on the COI mitochondrial DNA sequences using beast v2.4.7 (Bouckaert et al., 2014). One high‐quality COI sequence of each of the 1,360 flower‐visitor species was selected for the analysis. Sequences under 500 base pairs were omitted from the final analysis, leaving 1,314 sequences. Our approach was based on the findings of Boyle and Adamowicz (2015), who investigated the utility of COI data for estimating phylogenetic community structure. They found that, in general, COI data will estimate the relative genetic distances between pairs of co‐occurring species very well. Nonetheless, since the evolution of COI is subject to strong functional constraints (Pentinsaari, Salmela, Mutanen, & Roslin, 2016), it offers limited information for estimating the timing and divergence of deeper nodes in the phylogenetic tree, which could reduce the accuracy of COI phylogeny. In addition, phylogenies derived from single loci will always be subject to chance events and biases (Pamilo & Nei, 1988). Therefore, Boyle and Adamowicz (2015) recommended the use of an enforced backbone phylogeny—especially when dealing with data collected at a broader geographic scale and across more diverse taxonomic levels. Thus, we provided two sources of a priori information for the Bayesian analysis. First, we set the known monophyletic groups, and second, we provided a priori information on the divergence times of the deeper nodes (see Appendix S3, Supplemental information). In the analysis, branch lengths were allowed to vary under a lognormal relaxed clock model and the tree prior was set to the Yule model. The model was run for 500 million iterations, with samples taken after each 50,000 iterations. The function *bModelTest* in the package *BEAST* was used to identify the best substitution model given the data. beast analyses were run at the IT Center for Science Ltd. (CSC, http://www.csc.fi). We used tracer v1.6 (Rambaut, Suchard, Xie, & Drummond, 2014) to assess whether the likelihood trace of the run had converged to a stable equilibrium and to verify that ESS values for all parameters were >200. FigTree (http://tree.bio.ed.ac.uk/software/figtree/) was used to visualize and edit the phylogenetic tree.

### 2.9 Phylogenetic diversity measures

To characterize the phylogenetic diversity of a community, we used Faith's (1992) phylogenetic diversity (PD). This metric summarizes the total branch length among all taxa in a particular community, thus providing a convenient summary measure of each site. We calculated PD for all flower‐visitor communities using the package *picante* (Kembel et al., 2010) in r. Here, we used PD residuals to identify regions where PD is higher or lower than would be expected given species richness, and PD residuals were then used for further analyses. Areas of particularly high or low PD residuals could reveal information about the underlying structures of the flower‐visitor communities and indicate if these are taxonomically clustered or overdispersed (Forest et al., 2007; Rodriques, Brooks, & Gaston, 2005; Voskamp, Baker, Stephens, Valdes, & Willis, 2017). Unusually high PD residual values are detected in taxonomically overdispersed communities and could be the result of the existence of old lineages (Rodriques et al., 2005). At the other extreme, unusually low PD residual values indicate taxonomically clustered communities and are more likely to occur in areas with more recent speciation events (Davies & Buckley, 2011). A priori, we expected more phylogenetically diverse communities to consist of more functionally diverse taxa, likely to reflect more reliable and thereby augmented ecosystem functioning under rapidly variable arctic conditions (Kankaanpää et al., 2018). To relate phylogenetic diversity to seed set success of *Dryas*, we compared PD residuals to the difference in seed set success between the inside and outside of the pollinator exclosures (as reflecting the functional contribution of the local flower‐visitor community).

To examine the phylogenetic composition of flower‐visitor species in each community, we calculated the mean pairwise phylogenetic distance (MPD) for each community with package *metrictester* (Miller, Farine, & Trisos, 2017) in r. MPD indicates the mean pairwise phylogenetic distance separating taxa in a particular community (Webb, Ackerly, McPeek, & Donoghue, 2002). In these flower‐visitor communities, some species are rare while others are very abundant. Therefore, we focused on the abundance‐weighted measure of MPD, which is equal to the MPD among species weighted by the number of individuals of each species in a community. We calculated a specific version on abundance‐weighted MPD which only accounts for the interspecific phylogenetic distances (inter MPD, Miller et al., 2017). A priori, we expected higher MPD in communities closer to glacial refugia, where more genetic variation should have been retained over periods of climatic instability (Abbott et al., 2000; Hewitt, 2000). To examine the consistency between genetic diversity patterns in arthropods and plants, MPD values were compared to the genetic diversity measures in 17 alpine–arctic plant species (Eidesen et al., 2013).

To reveal the phylogenetic relatedness of flower‐visitor species among communities, we used function *comdistnt* in package *picante* (Kembel et al., 2010) in r. This metric represents the among‐community equivalent of mean nearest taxon index (MNTD, Webb, Ackerly, & Kembel, 2008; Webb et al., 2002), that is, a pairwise measure of phylogenetic β‐diversity among communities: the average phylogenetic distance to the most similar taxon in the other community for taxa in two communities. To describe patterns of similarity across sites, we used the pairwise values of phylogenetic distances to construct a dendrogram clustering communities based on their phylogenetic similarity using function *hclust* in package *picante* (Kembel et al., 2010) in r.

### 2.10 Testing for simultaneous associations

Since we explicitly dealt with spatial patterns across the Arctic, much of our inference builds on patterns of pairwise similarities in one metric compared to pairwise similarities in another (e.g., pairwise floristic similarity versus pairwise faunistic similarity). To test the significance for such patterns, we have used Mantel tests (above). Yet, spatial patterns in one metric may be associated with spatial patterns in another as due to a confounding third association. In particular, we will be interested in pinpointing the effect of whether differences in the exact *Dryas* species sampled at different site were reflected in differences in the flower‐visiting insect community. What we should therefore explicitly test for is the effect of similarities in space, including (a) the effect of space as such (basic “isolation by distance”); (b) the effect of resource similarity (the effect of exact *Dryas* species; if same species: similarity = 1; if different species: similarity = 0; if one of the two species is shared: similarity = 0.5); (c) the effect of climatic similarity etc.; and the effect of potential associations (a–c) on other patterns of key interest. To evaluate whether the association between focal metrics was confounded by the impact of other factors (pairwise geographic distance, pairwise similarity in temperature or in precipitation, or pairwise similarity of the resource basis, sensu *Dryas* species), we used partial Mantel tests, which tests partial correlation of two matrices conditioned on the third matrix. Denoting the two matrices to be compared by A and B, and the matrix to be controlled for by C, a partial Mantel test is implemented as a basic Mantel test of matrices A’ and B’, where A’ is the residual matrix of a regression of A on C, and B’ is the residual matrix of a regression of B on C. The partial Mantel tests were implemented in package *vegan* (Oksanen et al., 2016) in r. To test for the significance of the association, we used 999 random permutations of the matrices, with the *p*‐value identifying the fraction of randomizations showing an *r*‐value equal to or more extreme than the observed one. Hence, *p*‐values ≤0.025 and ≥0.0975 were deemed significant.

## 3 RESULTS

In total, we sampled 31,345 arthropods using the sticky flower mimics. Of these, we successfully sequenced and identified 13,681 individuals, detecting 1,360 BINs (for phylogenetic tree, see Figure 3; for a complete list, see Table S6, Supplemental information). Out of the 1,360 arthropod BINs detected, 488 were identifiable to a morphological species, 912 were identified to genus level, and 1,360 were identified to family level. In addition, 1,319 of the taxa matched to a previously known BIN in BOLD.

Overall, Diptera and Hymenoptera were the most abundant visitors of *Dryas*, representing 93.3% and 3.1% of all the pollinators sampled, respectively (Figure 2). The families most abundant across the sites were Chironomidae and Muscidae, accounting for 19.8% (*SE* ± 12.6%) and 19.4% (*SE* ± 9.1%) of the individuals, respectively. Yet, the flower‐visitor communities differed among sites in terms of both species richness and abundance (Figure 2). These overall differences reflected substantial variation in community composition and species abundances between sites, with pairwise faunistic similarity decreasing with an increasing distance between sites (Mantel test *r *=* *−0.47, *p *=* *1; for partial relationships, see Table S4, Supplemental information).

### 3.1 Environmental drivers of flower‐visiting species richness across the Arctic

**Figure 4:**
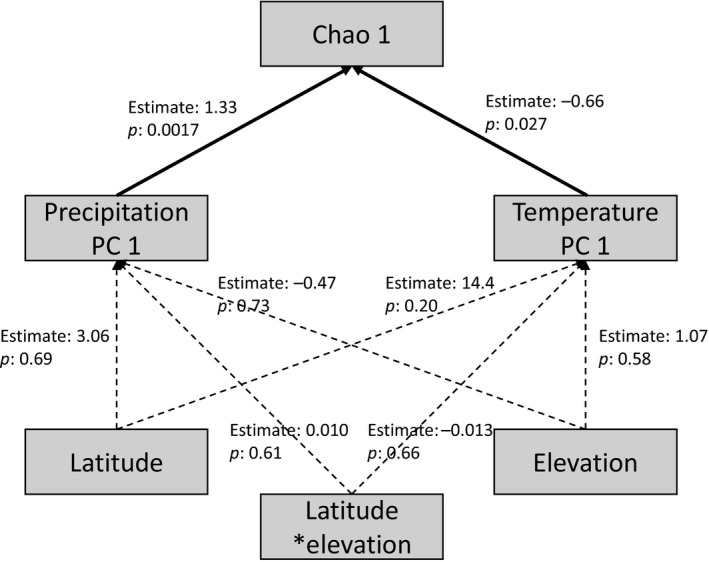
Direct and indirect effects of latitude, elevation, precipitation and temperature on the species richness of flower visitors (Chao 1 index). The figure shows a structural equation model (SEM) of the effects of latitude, elevation, the interaction between latitude and elevation, and the first principal components of precipitation and temperature metrics on local species richness, estimated by the Chao 1 index. The solid and dashed lines represent significant and non‐significant effects, respectively. The individual factor loadings of the PCs are shown in Tables S1 and S2 (Supplemental information)

Individual sites differed widely in climatic conditions, ranging from very cool to relatively warm sites (average temperature −14.9°C to −2.0°C, see Table S7, Supplemental information), and from arctic desert to relatively moist sites (annual precipitation 120 mm to 2,000 mm, see Table S7, Supplemental information). Overall, species richness, as characterized by the Chao 1 index, rose with increasing precipitation and with increasing temperature (as characterized by Precipitation PC 1 and Temperature PC 1, respectively; Figure 4). In particular, species richness increased with a general increase in the precipitation of the area and with warmer winter conditions. The latitude and elevation of the study site, and the interaction between the two, did not have any detectable effect on precipitation or temperature of the study site (Figure 4). Importantly, while patterns of climatic similarity were partly reflected in patterns of faunistic similarity, controlling for this association only marginally changed the association between faunistic and floristic similarity (Table S4, Supplemental information). Thus, climate comes with an impact on species richness (Figure 4), but the identity of the species making up this total is not dictated by climate per se (Table S4, Supplemental information).

### 3.2 The effect of Dryas species on patterns of similarity

Pairwise patterns in resource similarity (*Dryas* species) were congruent with patterns in floristic similarity (Mantel test: *r = *0.45, *p *=* *0.001) and faunistic similarity (Mantel test: *r = *0.43, *p *=* *0.001). However, this association was much weaker than the association between floristic and faunistic similarity as such: when the apparent association between faunistic similarity and resource similarity was controlled for their joint association with floristic similarity, they were no longer significantly congruent (Partial Mantel test: *r = *0.16, *p *=* *0.058; Table S4, Supplemental information).

In itself, pairwise patterns in resource similarity were associated with patterns in geographic distance as between‐site pairs (Mantel test: *r = *−0.54, *p *=* *1), with precipitation dissimilarity (Mantel test: *r = *−0.44, *p *=* *0.99) and with temperature dissimilarity (Mantel test: *r = *0.41, *p *=* *1, respectively; Table S4, Supplemental information). In other words, sites with different *Dryas* taxa were also further from each other and more dissimilar in terms of their abiotic environment than sites with the same taxon, a pattern consistent with different ranges in different *Dryas* taxa (e.g., Figure 2).

### 3.3 The effects of vascular plant community on flower‐visitor community

Patterns of floristic similarity and faunistic similarity were highly congruent across the study area. Overall, sites with a higher overlap in their vascular plant communities were also characterized by a higher overlap in terms of flower‐visiting arthropods (Mantel test, *r *=* *0.72, *p *<* *0.001; Figure S8, Table S4, Supplemental information). While part of this pattern could be traced to an effect of similarity in *Dryas* species on similarity in faunistic similarity (Mantel test: *r *=* *0.43, *p* = 0.001), the association between floristic similarity and faunistic similarity was substantially stronger (Mantel test: *r *=* *0.72, *p* = 0.001), and the latter pattern prevailed when corrected for the former (partial Mantel test, *r *=* *0.65, *p* = 0.001).

### 3.4 Within‐community phylogenetic diversity and its functional consequences

As expected, higher species richness was associated with a higher phylogenetic diversity of flower‐visitor communities across the Arctic (Figure S9B, Supplemental information). PD residuals, reflecting higher phylogenetic diversity than expected on the basis of species richness alone, were most positive (i.e., PD values higher than expected from species richness alone) in Kevo, Finland, and at Toolik Lake, Alaska, USA. The most negative PD residual values were detected on Coats Island, Canada, and at Furka, Switzerland.

**Figure 5:**
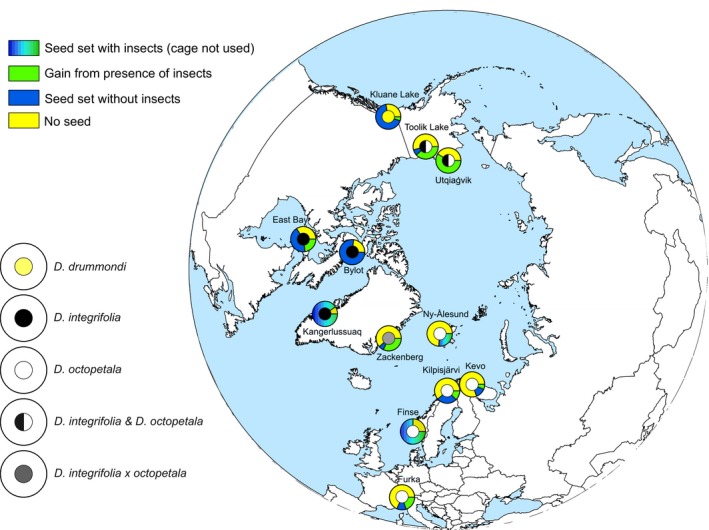
Seed set of *Dryas* in different parts of the Arctic. The blue, green and yellow parts of the pie charts represent the level of autogamy, the effect of pollination and the fraction of unsuccessful seed heads, respectively. A gradient between blue and green is used in sites where the level of autogamy was not successfully measured due to mishaps in the operation of pollinator exclosures. The identity of the *Dryas* taxa examined at the respective sites is shown by the colour of the centre of the pie charts (see legend in figure)

Seed set of *Dryas* showed great variation across the Arctic (Figure 5). On average, 56.4% ±18.1% of the seed heads produced seeds, with site‐specific values ranging from 29% to 90%. Similarity in seed set among sites was not attributable to the distance between the sites compared (Mantel test: *r = *−0.02, *p *=* *0.587) or to any other relationship tested for (e.g., climate; Table S4, Supplemental information). Contrary to expectations, the phylogenetic diversity (PD residuals) of the flower‐visitor community had no detectable impact on arthropod‐induced pollination (i.e., the difference in seed set success between the inside and outside of the pollinator exclosures; Figure S9C, Supplemental information).

**Figure 6:**
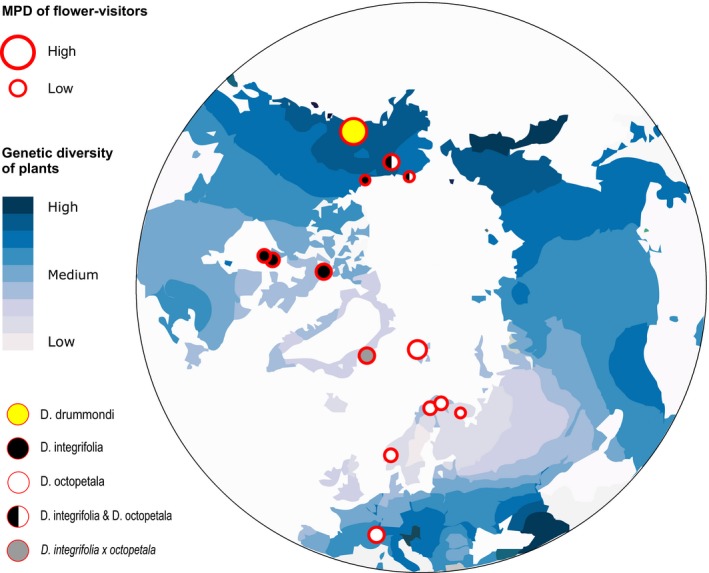
The consistency between mean pairwise phylogenetic distance (MPD) of the *Dryas* flower‐visitor communities across the Arctic and extrapolated genetic diversity in 17 arctic–alpine plant species (from Eidesen et al., 2013). The size of each circle represents the magnitude of the MPD value for each flower‐visitor community. The dark blue areas represent the highest overall plant genetic diversity, while the light yellow areas represent the lowest diversity. The identity of the *Dryas* taxa examined at the respective sites (Table 1) is shown by the colour of the centre of the circles (see legend in figure)

In terms of mean phylogenetic distance between flowervisitors (MDP), values were highest in some areas of Beringia (like Kluane Lake, Canada (751.4) and lowest in MacKenzie, Canada (412.0) Figure 6). Thus, the species composition in Kluane Lake visitor community was phylogenetically more diverse compared to other pollinator communities surveyed and consisted of phylogenetically less closely related species, while the MacKenzie visitor community consisted of more closely related species than communities at other sites.

### 3.5 Between‐community phylogenetic diversity

**Figure 7:**
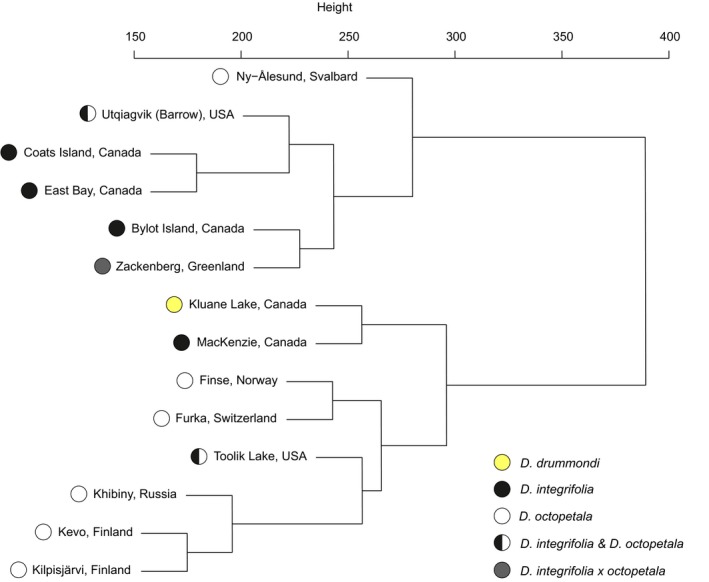
Dendrogram clustering *Dryas* flower visitor communities based on their phylogenetic similarity, here measured by mean nearest taxon distance (MNTD) values among all community pairs. Here, the colour of the circles shows the identity of the *Dryas* taxon (or taxa) examined at each site (Table 1)

In pairwise comparisons, MNTD values varied widely among flower‐visitor communities (from 174.3 to values more than twice as high, 377.0). Thus, some community pairs were much more similar to their phylogenetic composition than were others. The main division line appeared between Europe extending into Beringia on the one hand and North America proper on the other. Thus, all European communities clustered with each other and with one Alaskan and two Canadian communities. In contrast, one Alaskan community grouped together with three Canadian communities, the Greenland community and the Svalbard community (Figure 7).

## 4 DISCUSSION

*Dryas* flowers attract a vast diversity of arthropod visitors, showing that *Dryas* forms a widely used floral resource across the Arctic. From this single plant taxon, we sampled, successfully sequenced and identified a total of 1,360 different arthropod species by matching them to BINs in BOLD. In most of the regions examined, insects belonging to the orders Diptera and Hymenoptera were the most abundant visitors to *Dryas* flowers. Overall, the richness of visitors appeared driven by climatic factors associated with temperature and precipitation. The community structure of flowervisitors was closely linked to that of vascular plant species across the Arctic, while the geographically separate regions showed variable levels of taxonomic and phylogenetic distinctiveness. The phylogeographic structure found among the flower visitor communities seemed reflective of geological history and mirrored trans‐arctic patterns detected in plants. Seed set success varied widely among sites with little variation attributable to pollinator species richness. This pattern seems reflective of idiosyncratic associations caused by the dominant pollinator taxa varying between the areas. Below, we examine each of these findings in turn, noting that *Dryas* taxon in itself seemed to have no detectable imprint on any of the patterns discussed (Figures 2 and 5, 6, 7; Supplemental Figures S8 and S9; Table S4).

### 4.1 How does the structure of the flower‐visitor community vary in space?

All natural ecological communities are known to be characterized by a few taxa dominating the species abundance distribution, followed by many rare species (Fisher, Corbet, & Williams, 1943; McGill et al., 2007; Preston, 1948). In our study, local flower*‐*visitor communities were dominated by widely different flower‐visitor taxa (Table S5), with no single taxon retaining a key role in the pollinator community. A similar pattern has been observed in other flower‐visitor communities (e.g., Moeller, 2005; Potts, Vulliamy, Dafni, Ne'eman, & Willmer, 2003). As arctic pollinators vary substantially in their ability to carry pollen (Nielsen & Schmidt, 2013; Schmidt et al., 2016), their abundance alone is not enough to explain their efficiency as pollinators, and relative abundance is thus a poor proxy of functional importance. Overall, the structure of local flower‐visitor communities varied substantially in terms of both taxonomic and phylogenetic descriptors. While our study spanned multiple taxa within the genus *Dryas* (Table 1), the plant taxon as such had an only minor imprint on the composition of the arthropod community (Figures 2, 6 and 7; Figures S8 and S9, Table S4, Supplemental information). Species richness was found to be highest in the Northern European flower‐visitor communities (Figure 2, Table 1). These particular communities shared a large proportion of the visitor species detected, indicating a common source region for species in these communities during post‐glacial colonization. Overall, the pollinator communities at our study sites showed high α‐diversity (local species richness; Figure 2), even though sampling at each site was spatially and temporally limited to a relatively small area and to a short sampling period. The species turnover between sites was high, and the most abundant pollinator taxa varied among areas. High species turnover seems to contrast with Rapoport's rule (Stevens, 1989), which proposes that species range increases with latitude. Such a general pattern would imply low turnover of species at high latitudes, whereas our findings reveal high β‐diversity (site‐to‐site variation in community composition) in the Arctic. In this context, we note that we used a DNA‐based species concept—the BINs of Ratnasingham and Hebert (2013). That the BINning algorithm matches species limits established by other criteria has been verified by Ratnasingham and Hebert (2013) for multiple taxa, for Canadian insects by Hebert et al. (2016) and for the arctic fauna by Wirta et al. (2016). Thus, we are confident that the pattern of high species turnover among sites is real and matched by patterns in the richness of species defined by “traditional” criteria.

In terms of phylogenetic diversity within communities, PD and species richness were tightly linked—as previously detected in global studies for mammals (Davies & Buckley, 2011), terrestrial birds (Voskamp et al., 2017) and amphibians (Fritz & Rahbek, 2012). Residuals from this relationship (i.e., PD residual) did not show any clear geographic pattern. In birds, islands and isolated areas have been identified as areas with high PD residuals (Voskamp et al., 2017) indicating taxonomically overdispersed communities, whereas for amphibians and mammals, they tend to be the regions of low PD residuals (Davies & Buckley, 2011; Fritz & Rahbek, 2012) suggesting taxonomically clustered communities. Across the Arctic, no such patterns were found. Hence, local phylogenetic diversity seemed moulded by other factors, such as the geological history of arthropod recolonization.

In contrast to species richness, the mean pairwise phylogenetic distance (MPD) values were highest in the Canadian pollinator community, Kluane Lake (Figure 6), suggesting that this community consists of phylogenetically diverse pollinator species. Interestingly, the next highest MPD values after Kluane Lake were recorded from the North Atlantic islands, Greenland and Svalbard, where species richness was found to be lowest. This pattern could be a consequence of several source regions during post‐glacial colonization, thus leading to a phylogenetically diverse community despite low species richness (Alsos et al., 2015; Eidesen et al., 2013). How these patterns relate to likely assembly processes shaping communities of both arctic flower visitors and plants is discussed further below.

### 4.2 How are arctic flower‐visitor communities assembled?

Arctic flower‐visitor communities appeared to deviate from the generally recognized pattern of decreasing biodiversity with increasing latitude (Hillebrand, 2004; Pianka, 1966). This result may arise from the fact that our latitudinal span was relatively small (61 °N–79 °N, Furka 47 °N; Table 1) and from the fact that the sampling design focused on sites characterized by similar vegetation at a small scale (sites with *Dryas*). Unlike latitude, several abiotic factors were associated with spatial variation in community structure. Overall, precipitation explained the variation in species richness of flower visitors across sites (Figure 4; Table S3, Supplemental information). That precipitation has a major impact on a majority of arctic arthropods is quite conceivable, given that much of the Arctic is characterized by conditions typical of half‐desert or true arctic desert (Laity, 2008).

The pattern of community structure in flower visitors was similar to that of vascular plant species across the Arctic (Figure S8, Supplemental information). Again, we found no detectable imprint of the specific *Dryas* taxa included in the comparison (Table S4, Supplemental information). The positive association between faunistic and floristic similarities (Figure S8, Supplemental information) suggests that the two communities have been moulded by the same biogeographical processes, in particular by similar refugial and post‐glacial history (a pattern robust to controls for confounding patterns; Table S4, Supplemental information). To evaluate whether this interpretation holds true, we compared our results to the distribution of genetic diversity and the borders for gene flow found in vascular plants (Eidesen et al., 2013; Stewart et al., 2016). In both studies, plant genetic diversity was found to be highest in “Beringia” (Hultén, 1937), that is, the area around the Bering Strait, a region that was never glaciated during the Pleistocene (Dyke 2004). Beringia is a hotspot for species diversity and endemism, and is known to have served as a major glacial refugium for arctic flora and fauna (Cook et al., 2005; Hewitt, 2000; Hultén, 1937). In addition, plant genetic diversity gradually decreased into the area that was under ice during the last glacial maximum (20,000 years ago, Frenzel, 1968), which further supports the existence of a large, long‐standing refugium in Beringia (Hultén, 1937). Our results are consistent with this major pattern in plants, as the MPD values of arthropod communities were highest close to Beringia (Kluane Lake, Canada) and lowest in the area that was under glacial cover during the last glacial maximum (Figure 6).

The strongest barriers for gene flow on vascular plant species in the circumpolar Arctic have been identified as the Arctic and Atlantic Oceans, the Greenlandic ice cap, the Urals and lowland areas between southern mountain ranges and the Arctic (Eidesen et al., 2013). Based on our findings, the Arctic and Atlantic Oceans could act as strong barriers for dispersal of flower‐visitor species, too. Phylogenetic similarity grouped the European communities with one Alaskan and two Canadian communities (Figure 7). As the second main cluster, one Alaskan community grouped together with three Canadian communities, with the Greenland community and with the Svalbard community. Furthermore, the three Northern European communities were clustered together in the analysis, adding evidence that those communities share several phylogenetically related species (Figure 7).

The patterns described above are consistent with a biogeographical pattern in which the flower‐visitor species migrated east and west from the glacial refugium in Beringia. Thus, historical events, such as colonization patterns after the last glacial maximum, the ice extent during the last glacial maximum and the locations of glacial refugia, have shaped the current spatial distribution of species in the Arctic. A similar biogeographical pattern has been reported in prior studies, for example, springtails (Collembola, Ávila‐Jiménez & Coulson, 2011) and in a vascular plant species *Saxifraga oppositifolia* (Abbott 2006). Yet, in contrast to our results, springtails in Greenland were more closely related to those in the European Arctic than to springtails in the Canadian Arctic (Ávila‐Jiménez & Coulson, 2011). Akin to patterns in springtails (Ávila‐Jiménez & Coulson, 2011), the single most important source region for the plants of both East Greenland and Svalbard was found to be Northwestern Russia (Alsos et al., 2015). In our results, the high MPD values detected in Greenland and Svalbard could support the existence of several source regions for those areas after the LGM. Therefore, more comprehensive sampling design including flower‐visitor communities in northern Russia could have provided a better overview of the phylogenetic relatedness of flower*‐*visitor communities between the North Atlantic islands and the European Arctic, as well as between Beringia and Northern Europe. Nonetheless, large‐scale post‐glacial colonization typically occurs from more than one source region, which are often not the closest potential source regions (Alsos et al., 2015).

### 4.3 Is community structure reflected in function?

We expected that higher taxonomic and phylogenetic diversity in a certain flower‐visitor community would be associated with larger functional diversity. In a previous study conducted at a local scale, one muscid fly species, *Spilogona sanctipauli,* was found to dominate the functioning of a pollinator community at Zackenberg, Greenland (Tiusanen et al., 2016). In that study, seed set in *Dryas* was found to increase with the abundance of *S. sanctipauli*. This finding identifies species identity rather than overall species richness as the main driver of arctic ecosystem functioning. In our current results at the global scale (but including one of the local samples of Tiusanen et al., 2016), the success in seed set of *Dryas* was found to vary widely among sites, as likely due to, for example, abiotic conditions or phenotypic, genetic or taxonomic differences of plants between the sampling locations. By contrast, little variation seemed attributable to the identity of the local *Dryas* taxon (Figure 5; Figure S9A, Table S4, Supplemental information) or to flower‐visitor community structure. All of the flower‐visitor communities studied were found to be dominated by different species, and no particular taxon is likely to sustain functioning across all sites. This suggests that the cumulative number of species required to sustain pollination across the Arctic is higher than the number of species needed at any one site. Other studies have also shown that the cumulative number of species needed for pollination increases with spatial scale (Cardinale et al., 2011; Isbell et al., 2011; Tilman et al., 2014; Winfree et al., 2018). In addition to pollinator taxa, further contributors, such as variation in abiotic conditions at both small and larger scales, are naturally likely to contribute to variation in seed set at the global scale.

Even though arthropod flower visitors increased the seed set of *Dryas* at all locations studied, the magnitude of the effect varied largely. One potential explanation for this is local differences in breeding systems among different *Dryas* strains. For instance at Utqiaģvik (Barrow), *Dryas* did not produce any seeds at all if the flower visitors were excluded, whereas the local seed set success in the presence of flower visitors was 65.6% (Figure 5). At Kevo and Kilpisjärvi, Ny‐Ålesund and Bylot Island, the gain from visits by arthropods amounted to less than 10% (with seed set success in the presence of flower visitors varying between 28.8% and 73.3%). These wide ranges suggest that some *Dryas* strains may have adapted to varying pollinator availability in the Arctic by evolving high levels of autogamy and, thus, reduced pollinator dependence. It is also possible that some increase in seed set with visits by arthropods derives not only from an effect on outcrossing rate, but also from some increase in autogamy induced by flower visitors crawling around in the flowers. Importantly, the difference was not attributable to the *Dryas* species as such (Table S4, Supplemental information).

### 4.4 Conclusions

Overall, the use of DNA barcodes allowed us to overcome the taxonomic impediment common in arthropod diversity studies and to address ecological patterns involving thousands of taxa, each hard to identify. DNA barcodes also contain useful information on species relatedness, allowing us to simultaneously assess how phylogenetically diverse communities are formed on a single plant resource under different biogeographical and abiotic conditions. Furthermore, the phylogenetic community analyses allowed us to reveal the processes driving the flower‐visitor community assembly at a global scale, including the importance of historical factors and biogeographical patterns in the community assembly process in the Arctic. Overall, trans‐Arctic and high‐alpine patterns of community structure in flower‐visitor communities were found to be similar to those previously described for vascular plant species in cold, arctic–alpine habitats, and the phylogeographic structure found seemed reflective of geological history. Taken together, these insights provide a new understanding of community assembly processes acting across space and in time.

## AUTHOR CONTRIBUTION

The research was designed by M.T., B.H. and T.R. The sampling was conducted by T.A., A.A., E.D., J.G., C.K., R.B.L., M.J.J.E.L., R.P., K.R., S.T.S., F.S.‐G., J.Š., I.S., M.T., C.U., S.W., P.W. and Y.Z. The molecular work was performed by M.T. and the laboratory of P.H. The data analyses were performed by M.T., T.H. and TR. The manuscript was written by M.T., T.H. and T.R., with contributions by all authors.

## Data Availability

The complete sequence data set is available at dx.doi.org/10.5883/DS-POLARC.
