# Supplementary material for: Flower‐visitor communities of an arcto‐alpine plant—Global patterns in species richness, phylogenetic diversity and ecological functioning
Source: Mol Ecol. 2018 Dec 7;28(2):318–35. doi: 10.1111/mec.14932 (PMC6378624; doi:10.1111/mec.14932)
Supplement: Supplementary file 1 [file MEC-28-318-s001.zip › mec14932-sup-0001-Supinfo.docx]

**Supplemental Information for:**

**Flower-visitor communities of an arcto-alpine plant– global patterns in species richness, phylogenetic diversity and ecological functioning**

Mikko Tiusanen, Tea Huotari, Paul D.N. Hebert, Tommi Andersson, Ashley Asmus, Emma Davis, Jennifer Gale, Bess Hardwick, David Hik, Christian Körner, Richard B. Lanctot, Maarten J.J.E. Loonen, Rauni Partanen, Karissa Reischke, Sarah T. Saalfeld, Fanny Senez-Gagnon, Ján Šulavík, Ilkka Syvänperä, Christine Urbanowicz, Sian Williams, Paul Woodard, Yulia Zaika & Tomas Roslin

**Table of Contents:**

| **Figure S1** | Page 2 |
| --- | --- |
| **Figure S2** | Page 3 |
| **Table S1** | Page 4 |
| **Figure S3** | Page 5 |
| **Appendix B** | Page 6 |
| **Table S2** | Page 7 |
| **Figure S4** | Page 8 |
| **Table S3** | Page 9 |
| **Figure S5** | Page 10 |
| **Figure S6** | Page 11 |
| **Figure S7** | Page 12 |
| **Appendix C** | Page 13 |
| **Table S4** | Page 14 |
| **Figure S8** | Page 15 |
| **Figure S9** | Page 16 |


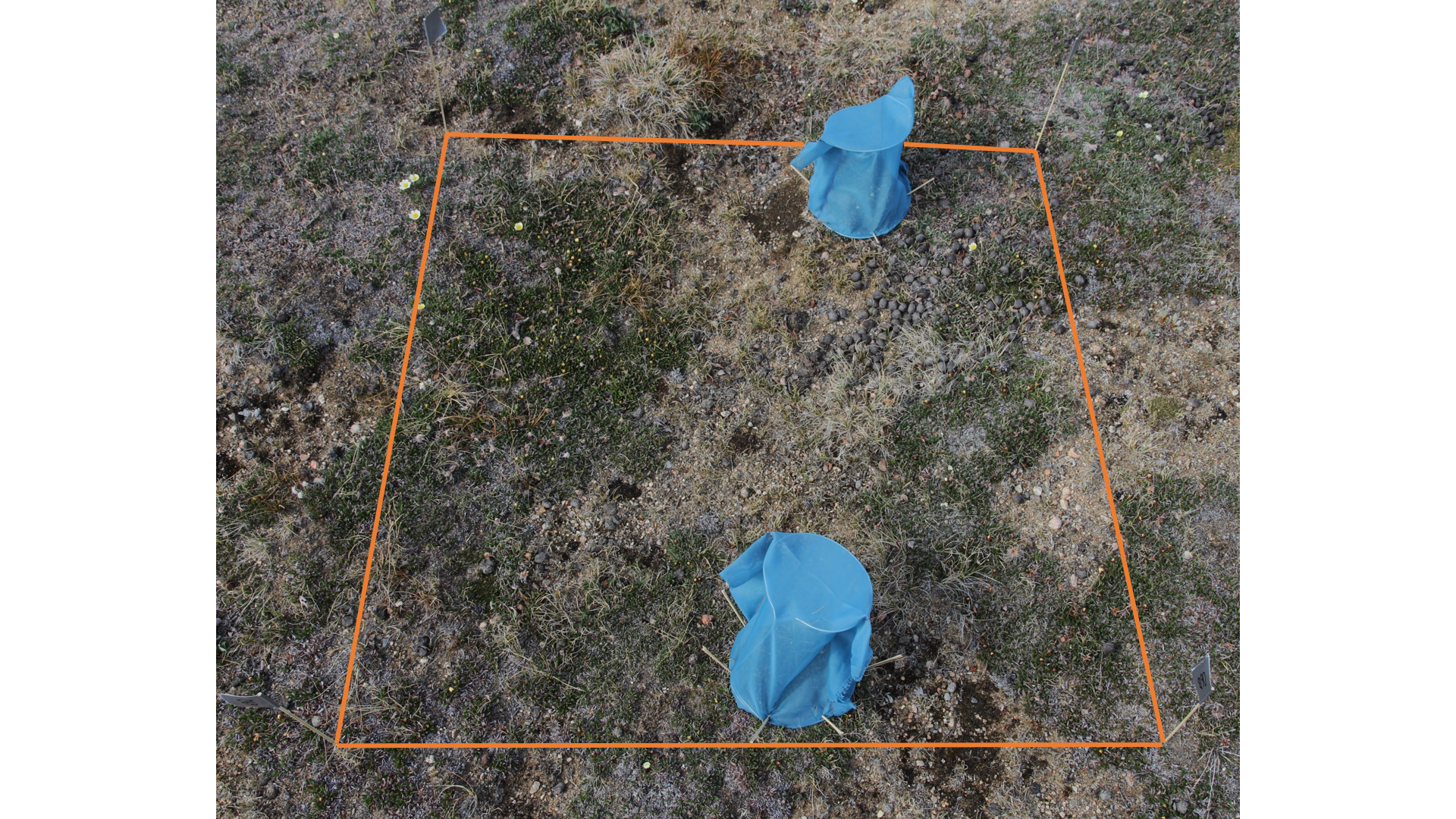


**Figure S1. Devices used to exclude insect visitors.** Sampling grids of 1m × 1m were marked with small flags made out of cocktail sticks and duct tape. To exclude pollinators from visiting selected flowers in each study square, we used two small tents placed in random locations within each grid. Twenty sticky traps (see Fig. S2) were placed within each sampling grid once half of the *Dryas* flowers had opened.

**
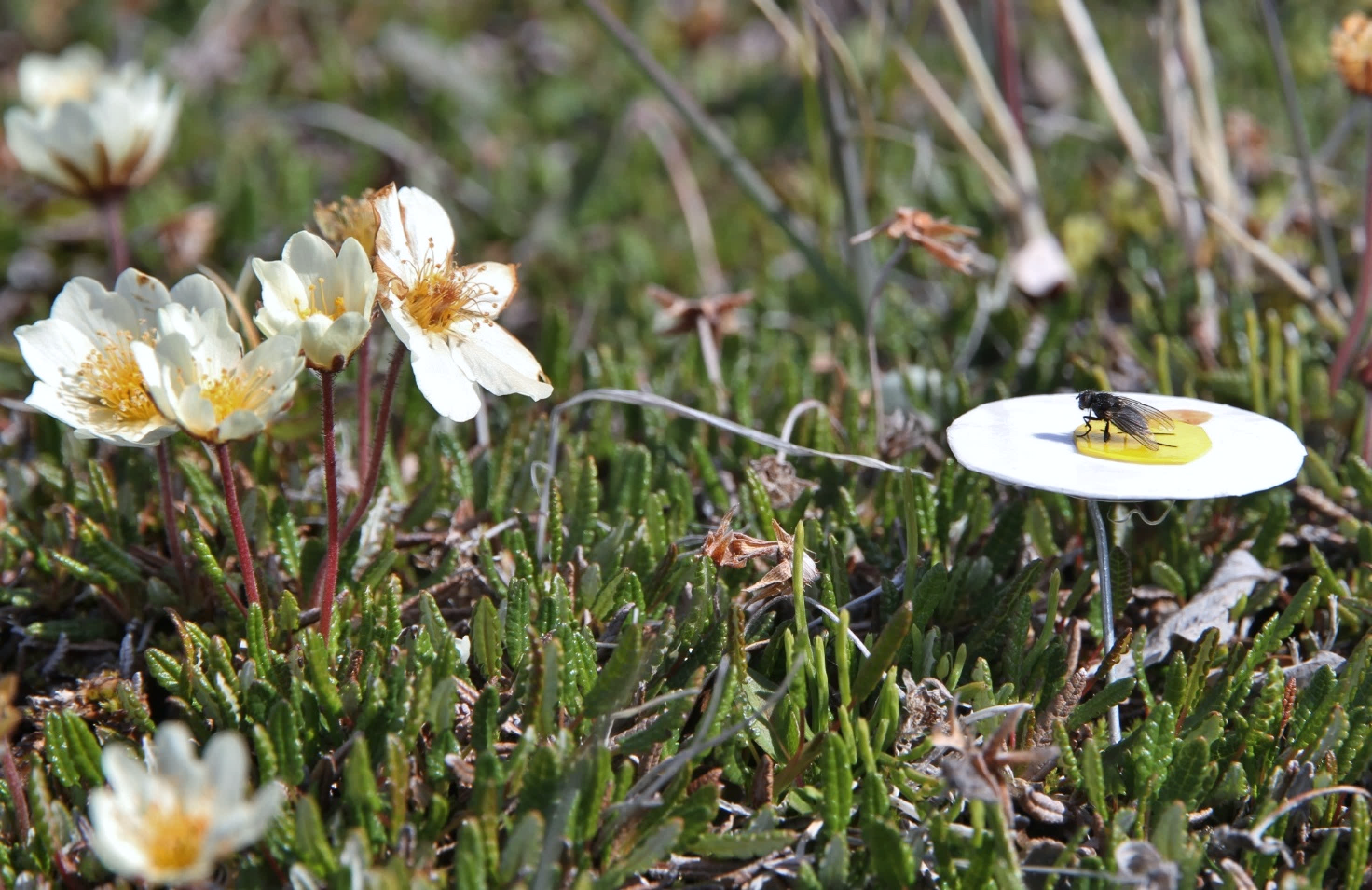
**

**
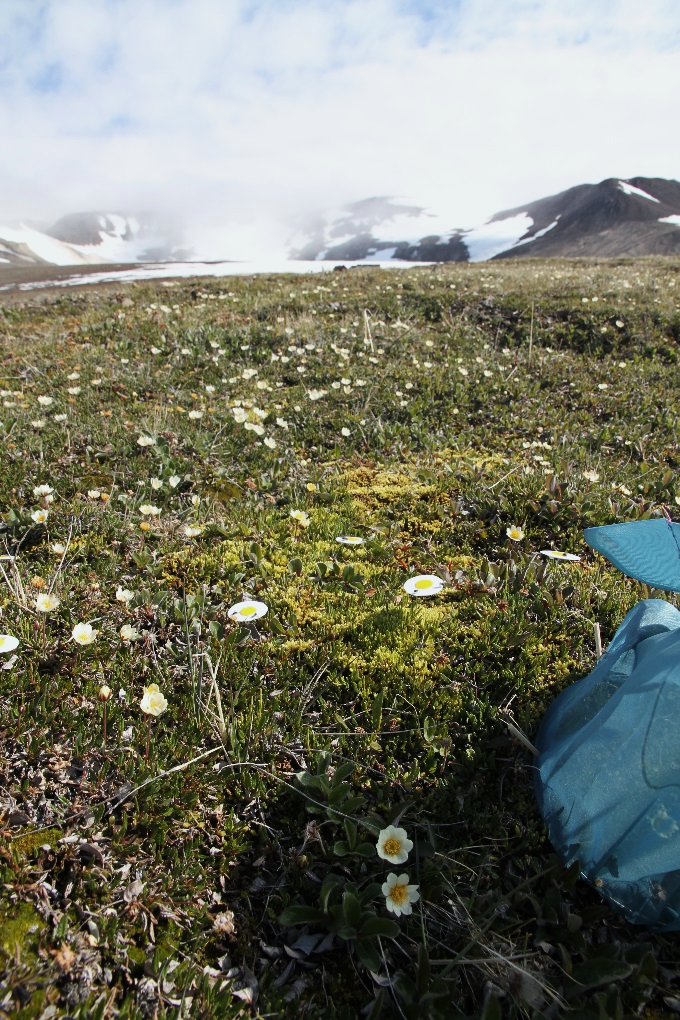
Figure S2. Sticky traps used to sample flower visitors.** Each trap was made of two circular pieces of sticky paper: a white piece (ø30 mm; made of Sticky Roll, Barrettine Environmental Health, Barrettine Group, Bristol, UK) to represent the petals and a yellow piece (ø8 mm; Yellow Sticky Board, Barrettine Environmental Health, Barrettine Group, Bristol, UK) to represent the stamen. For attachment, we used a “stem” made out of iron wire, stuck into the soil to a depth exposing the flower mimic level with natural flower heads.

**Table S1**. The compositions of the first three principal components of the composition of flower visitor samples in the pilot study by Visakorpi et al. (2014). Columns ‘PC 1’, ‘PC 2’ and ‘PC 3’ identify the first, second and third principal component, respectively. The first row contains information on the proportion of variance explained by the principal components, whereas the other rows contain information on the loadings of different insect groups in the principal components. Consistent with the section on Sampling of flower-visitors, data was transformed to flower-visitors/hour, thereby scaling observation periods of different time to the same unit. In addition, data on real flowers contained many observations of no pollinators at all as relate to some rather short observations periods (5–20 min). These observations were excluded from the PCA analysis, since a) the re-revised text (section Sampling of flower-visitors) contains a separate comparison of visitation rates per time unit to real vs sticky flowers (revealing no difference), and b) observations of no community are strictly uninformative for comparisons of community structure.

|  | PC1 | PC2 | PC3 |
| --- | --- | --- | --- |
| Proportion of variance explained | 0.25 | 0.19 | 0.08 |
|  |  | loadings |  |
| Anthomyiidae/Muscidae | -0.96 | 0.26 | 0.01 |
| Empididae | 0.27 | 0.96 | -0.02 |
| Syrphidae | -0.01 | -0.03 | -0.99 |
| Chironomidae | 0.04 | -0.05 | 0.13 |
| Hymenoptera | 0.00 | 0.00 | 0.01 |
| Lepidoptera | 0.00 | 0.00 | 0.00 |
| Other | 0.00 | 0.00 | 0.00 |

**A)**

**B)**

**Figure S3.** PCA ordination of the composition of flower visitor samples derived by real (black symbols) versus sticky flower mimics (red symbols) in the pilot study by Visakorpi et al. (2014). Shown are the first two principal components of community composition (*sensu* flower-visitors/hour; for the composition of these axes and factor loadings, see Table S1). Different markers (open circle, open square, cross, x-mark) represent four different sampling sites. The concentration of points around the origo in panel A) is resolved by magnification in panel B) (compare scale of axes). PCA1 mainly reflects variation in fly abundance, whereas PC2 was disproportionately affected by to two samples containing single individuals of Diptera: Empididae and no other insects (Table S1). All other samples – regardless of sampling method – are diagonally aligned in the plot, revealing no major separation by sampling method. We note that visitors come as integers, and that the smallest number observed is 1. Thus, the overrepresentation of real flowers on the right-hand side of the graph corresponds to single flower-visitors observed during short observation periods (e.g. 20 min).

**Appendix B.** Details of the DNA barcode analysis, DNA extraction, amplification and sequencing of flower-visitors.

For each specimen, DNA was first extracted from a small piece of tissue as described by Ivanova et al. (2006). Polymerase chain reaction (PCR) was then used to amplify the COI region from DNA extractions using primers LCO1490 and HCO2198 (Folmer et al. 1994). PCR reactions were performed with the following protocol in a total volume of 12.5 μl: 6.25 μl of 10% trehalose (Sigma), 2 μl of distilled water, 1.25 μl of 10X buffer (Platinum Taq, Invitrogen), 0.625 μl of 50 nM MgCl2 (Invitrogen), 0.125 μl of each 10 μM primer (primers listed below) and 0.0625 μl of 10 mM dNTPs (New England Biolabs), 0.06 μl of polymerase (5U/μl Platinum Taq, Invitrogen) and 2 μl of the DNA template. The PCR cycling conditions were as follows: 1 min in 94°C, followed by 5 cycles of 30 s in 94°C, 40 s in 45-50°C and 60 s in 72°C, then 30-35 cycles of 30 s in 94°C, 40 s in 51-54°C and 60 s in 72°C, ending with 10 min in 72°C. PCR products were then sequenced using Sanger sequencing technology.

References

Folmer, O., Black, M., Hoeh, W., Lutz, R. & Vrijenhoek, R. (1994) DNA primers for amplification of mitochondrial cytochrome c oxidase subunit I from diverse metazoan invertebrates. *Molecular Marine Biology and Biotechnology* **3**, 294-299.

Ivanova, N.V., de Waard, J.R. & Hebert, P.D.N. (2006) An inexpensive, automation-friendly protocol for recovering high-quality DNA. *Molecular Ecology Notes* **6**, 998-1002.

**Table S2**. The compositions of the first three principal component of the temperature PCA. Columns ‘PC 1’, ‘PC 2’ and ‘PC 3’ identify the first, second and third principal component, respectively. The first row contains information on the proportion of variance explained by the principal components, whereas the other rows contain information on the loadings of different temperature variables in the principal components.

| Temperature PCA | PC 1 | PC 2 | PC 3 |
| --- | --- | --- | --- |
| Proportion of variance explained | 0.53 | 0.37 | 0.08 |
|  |  | loadings |  |
| Annual Mean Temperature | 0.38 | -0.16 | -0.14 |
| Mean Diurnal Range (Mean of monthly (max temp - min temp)) | -0.07 | -0.41 | 0.55 |
| Isothermality (Diurnal range/Annual range) | 0.29 | -0.19 | 0.63 |
| Temperature Seasonality (standard deviation) | -0.37 | -0.22 | -0.07 |
| Max Temperature of Warmest Month | 0.08 | -0.48 | -0.13 |
| Min Temperature of Coldest Month | 0.40 | 0.07 | -0.15 |
| Temperature Annual Range (max temp. – min temp) | -0.32 | -0.31 | 0.06 |
| Mean Temperature of Wettest Quarter | 0.12 | -0.45 | -0.31 |
| Mean Temperature of Driest Quarter | 0.38 | 0.09 | 0.24 |
| Mean Temperature of Warmest Quarter | 0.17 | -0.43 | -0.27 |
| Mean Temperature of Coldest Quarter | 0.41 | 0.02 | -0.08 |

**Figure S4. Loadings of the temperature variables on the first two principal components of the Temperature PCA.** The labels on the vectors identify:

1) Annual mean temperature

2) Mean diurnal range (mean of monthly (maximum - minimum temperature))

3) Isothermality (diurnal range/annual range)

4) Temperature seasonality (standard deviation)

5) Maximum temperature of warmest month

6) Minimum temperature of coldest month

7) Temperature annual range (maximum – minimum temperature)

8) Mean temperature of wettest quarter

9) Mean temperature of driest quarter

10) Mean temperature of warmest quarter

11) Mean temperature of coldest quarter

**Table S3**. The compositions of the first two principal component of the precipitation PCA. Columns ‘PC 1’ and ‘PC 2’ identify the first, second and third principal component, respectively. The first row contains information on the proportion of variance explained by the principal components, whereas the other rows contain information on the loadings of different precipitation variables in the principal components.

| Precipitation PCA | PC 1 | PC 2 |
| --- | --- | --- |
| Proportion of variance explained | 0.85 | 0.13 |
|  | loadings | |
| Annual Precipitation | 0.38 | 0.00 |
| Precipitation of Wettest Month | 0.35 | -0.40 |
| Precipitation of Driest Month | 0.38 | 0.13 |
| Precipitation Seasonality (Coefficient of Variation) | -0.29 | -0.61 |
| Precipitation of Wettest Quarter | 0.36 | -0.34 |
| Precipitation of Driest Quarter | 0.38 | 0.17 |
| Precipitation of Warmest Quarter | 0.34 | -0.43 |
| Precipitation of Coldest Quarter | 0.35 | 0.34 |

**Figure S5.** Loadings of the precipitation variables on the first two principal components of the Precipitation PCA. The labels on the vectors identify:

1) Annual precipitation

2) Precipitation of wettest month

3) Precipitation of driest month

4) Precipitation seasonality (coefficient of variation)

5) Precipitation of wettest quarter

6) Precipitation of driest quarter

7) Precipitation of warmest quarter

8) Precipitation of coldest quarter

**Figure S6.** The study sites plotted against the first principal components of the Temperature PCA and Precipitation PCA, respectively.


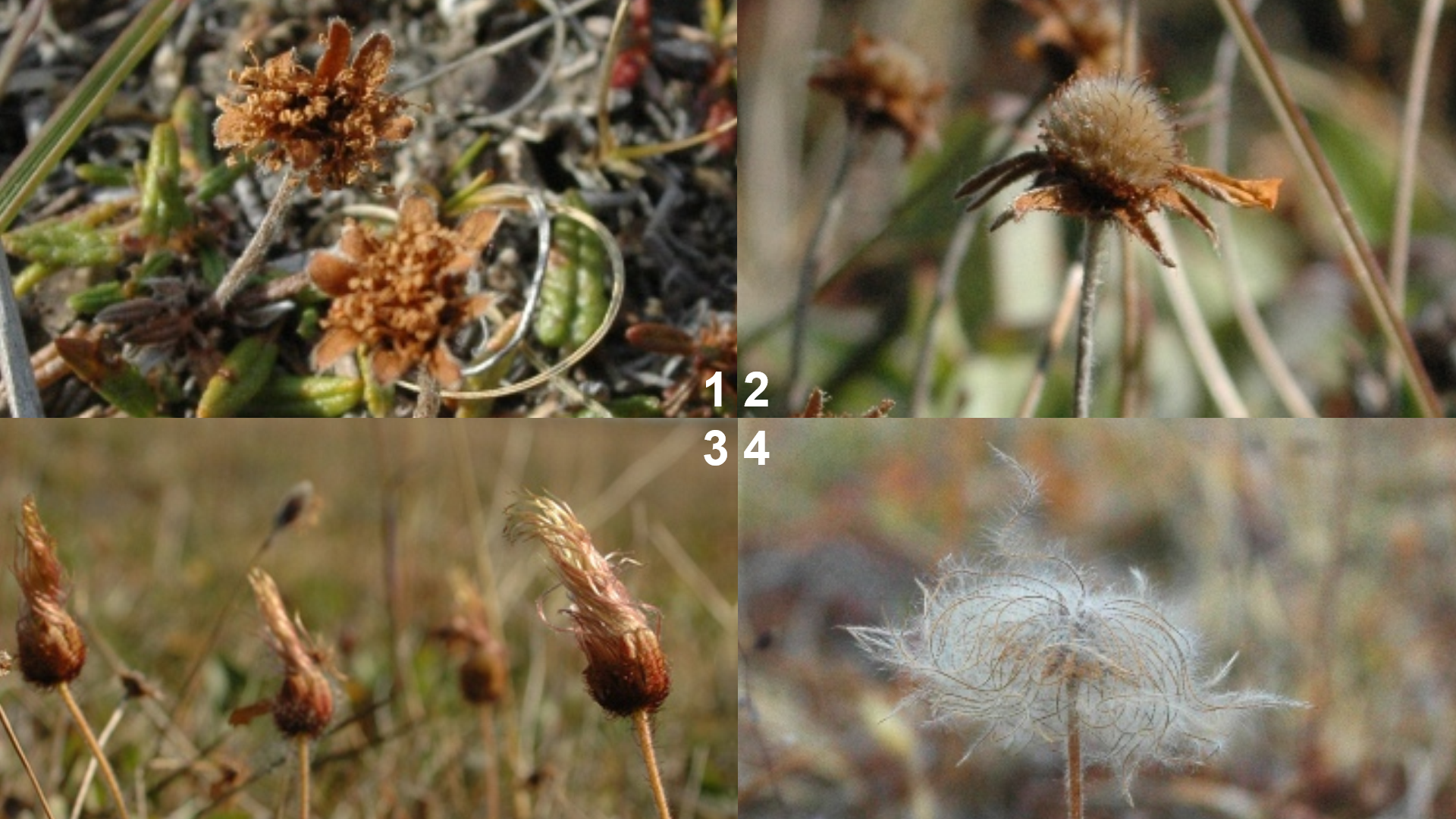


**Figure S7. Classification used to score the success of seed set in individual seed heads of *Dryas*.** At the end of the season, we counted all the seed heads of *Dryas* in the study squares and classified them into four categories:

1. Dry flower: no pistil or seed head visible

2. Flower with visible, round pistil but no clear seed head

3. Flower with clear, “torch”-like seed head

4. Open, umbrella-like seed head

Since fully-developed *Dryas* seeds possess a clear pappus, categories 3 and 4 were assumed to reflect viable seeds, while categories 1 and 2 were assumed to represent failed seed production.

**Appendix C.** Details of calibrating the Bayesian phylogeny.

First, we set all the orders, as well as the classes Insecta and Arachnida, subphylum Hexapoda and Dipteran suborder Brachycera as monophyletic groups. Second, we provided *a priori* information on the divergence times of the deeper nodes: Arachnida (494 Mya), Hexapoda (480 Mya), Collembola (439 Mya), Insecta (443 Mya), Hemiptera (344 Mya), Thysanoptera (344 Mya), Araneae (340 Mya), Hymenoptera (283 Mya), Diptera (272 Mya), Lepidoptera (232 Mya) and Coleoptera (297 Mya). A time estimate of the most recent common ancestor of all the orders (601 Mya) was used to calibrate the root of the tree. *A priori* divergence times were based on *TimeTree*, an online cross-study resource of divergence times (www.timetree.org; Hedges et al. 2015; Kumar et al. 2017). In the analysis, all divergence times were modeled as normal distributions with a mean and a standard deviation of 8 Mya.

References

Hedges, S.B., Marin, J., Suleski, M., Paymer, M. & Kumar, S. (2015) Tree of life reveals clock-like speciation and diversification. *Molecular Biology and Evolution*, **32**, 835–845.

Kumar, S., Stecher, G., Suleski, M. & Hedges, S.B. (2017) TimeTree: a resource for timelines, timetrees, and divergence times. *Molecular Biology and Evolution*, **34**, 1812–1819.

**Table S4.** Pairwise associations among sites in terms of floristic similarity, faunistic similarity, similarity in precipitation and temperature patterns (‘Temp similarity’ and ‘Prec similarity’, respectively; both derived through the PCA analyses explained in the main text), resource similarity (*Dryas* species; Table 1 in main text) and (Euclidean) distance between sites. To evaluate whether the association between two focal metrics was affected by the impact of another factor, the table includes both tests of associations between matrix pairs (Mantel tests) and tests controlling for associations with a third matrix (partial Mantel tests). To test the significance of the observed association, we used 999 random permutations of the matrices, with the *P*-value identifying the fraction of randomizations showing an *r*-value equal to or more extreme than the observed one. Hence, *P*-values ≤0.025 and ≥0.0975 were deemed significant and are indicated in bold face.

| Mantel tests | | | | |
| --- | --- | --- | --- | --- |
| First matrix | Second matrix |  | *r* | *P* |
| **Floristic similarity** | **Faunistic similarity** |  | **0.72** | **0.001** |
| **Floristic similarity** | **Resource similarity** |  | **0.45** | **0.001** |
| **Floristic similarity** | **Distance** |  | **-0.56** | **1.00** |
| Floristic similarity | Temperature |  | -0.17 | 0.935 |
| **Floristic similarity** | **Prec similarity** |  | **-0.34** | **0.985** |
| Floristic similarity | Functional similarity | | 0.194 | 0.197 |
| **Faunistic similarity** | **Resource similarity** |  | **0.43** | **0.001** |
| **Faunistic similarity** | **Distance** |  | **-0.47** | **1.00** |
| **Faunistic similarity** | **Temp similarity** |  | **-0.36** | **0.997** |
| **Faunistic similarity** | **Prec similarity** |  | **-0.3** | **0.993** |
| Faunistic similarity | Functional similarity | | 0.052 | 0.439 |
| **Resource similarity** | **Distance** |  | **-0.54** | **1.00** |
| **Resource similarity** | **Temp similarity** |  | **-0.44** | **0.99** |
| **Resource similarity** | **Prec similarity** |  | **-0.41** | **0.995** |
| Resource similarity | Functional similarity | | -0.2 | 0.94 |
| Functional similarity | Distance |  | -0.02 | 0.587 |
| Functional similarity | Temp similarity |  | 0.04 | 0.44 |
| Functional similarity | Prec similarity |  | -0.34 | 0.908 |
| **Distance** | **Temp similarity** |  | **0.42** | **0.006** |
| **Distance** | **Prec similarity** |  | **0.45** | **0.004** |
| **Temp similarity** | **Prec similarity** |  | **0.52** | **0.004** |
|  |  |  |  |  |
| Partial Mantel tests | | | | |
| First matrix | Secod matrix | Matrix controlled for | *r* | *P* |
| **Floristic similarity** | **Faunistic similarity** | **Distance** | **0.63** | **0.001** |
| **Floristic similarity** | **Faunistic similarity** | **Resource similarity** | **0.65** | **0.001** |
| **Floristic similarity** | **Faunistic similarity** | **Temp similarity** | **0.72** | **0.001** |
| **Floristic similarity** | **Faunistic similarity** | **Prec similarity** | **0.69** | **0.001** |
| **Floristic similarity** | **Faunistic similarity** | **Functional similarity** | **0.73** | **0.001** |
| Resource similarity | Faunistic similarity | Floristic similarity | 0.16 | 0.058 |

**Figure S8.** Floristic versus faunistic similarity among study sites (Mantel´s test, *r*=0.72, *p*<0.001). Shown is pairwise Jaccard similarity indices of plant vs flower-visiting-communities among all site pairs. Note difference in scale among axes. Symbol style identifies the identity of the *Dryas* taxa included in the pairwise comparison (see legend in figure). Note the lack of any imprint of *Dryas* taxon on patterns of similarity.

**Figure S9**. A) Gain from insect pollination (i.e. difference in seed set success between flowers out- versus inside of the flower-visitor exclosures) as a function of species richness. B) Faith’s phylogenetic diversity as a function of species richness C) Gain from insect pollination as a function of phylogenetic diversity D) Gain from pollination as a function of the residuals of phylogenetic diversity (i.e. deviations from the line in panel A). Here, symbol style represent the *Dryas* species present at the study site: Open circles, black dots, crosses, x-marks and open squares represent *D. drummondi, D. integrifolia, D. integrifolia & D. octopetala,* hybrid *D. integrifolia x D. octopetala* and *D. octopetala*, respectively (similarly as in the legend of Figure S8).

**C)**

**D)**

**B)**

**A)**
