# Supplementary material for: Flower‐visitor communities of an arcto‐alpine plant—Global patterns in species richness, phylogenetic diversity and ecological functioning
Source: Mol Ecol. 2018 Dec 7;28(2):318–35. doi: 10.1111/mec.14932 (PMC6378624; doi:10.1111/mec.14932)
Supplement: Supplementary file 1 [file MEC-28-318-s001.zip › mec14932-sup-0002-Appendix A.pdf]

Instructions

NB! If you visit your study site for only a shorter period of time, you may still collect valuable data: If this visit is scheduled for early or mid summer, we ask you to perform STEP 1 (without the placement of cages) and STEP 2. If the visit occurs in late summer, we ask you to simply estimate the number of seed heads in different categories (see below, STEP 3)

With this project, our goal is to understand the processes underlying the ecosystem services of the Arctic. As a model, we will use a fundamental ecosystem function of massive proportions: the pollination of Avens (genus *Dryas* in Rosaceae).

Your task is to sample the structure of the pollinator community, and to measure seed set by *Dryas*. By submitting these data to us, you will contribute to building the big picture: relating community structure to ecosystem functioning.

Each participating station should invest no more than 2-3 full person-work-days in the project. At each station, we ask you to implement a standardized protocol to first establish the sampling plots (STEP 1), then sample the local pollinator community of *Dryas* (STEP 2) and finally record the resultant seed set (STEP 3). All materials needed for implementing every stage will be supplied by the coordinator (bess.hardwick@helsinki.fi)

When you receive your kit, please check that it contains the following:

- Sampling forms 1-5
- Mailing instructions
- Ten exclusion cage frames
- Ten exclusion cage hoods, in plastic bag
- Thirty tent pegs
- 100 trap flower bases, on colour coded cardboard
- 100 trap flower centers, in plastic bag
- 20 flags, half colour coded
- 5 additional sticks to help with counting
- Envelope for collecting *Dryas* samples for leaf quality assessment
- Please keep the box, use it for returning the trap flowers to us

You will also need the following (not supplied):

- A 1 metre stick or a 1x1 metre frame
- A digital camera

STEP 1. *Half a day, depending on how easily Dryas vegetation is found. Today you will need the metal cage frames, fabric cage hoods, tent pegs and flag, as well as a 1-metre stick or frame and a camera, and sampling form 1.*

To control pollinator access to selected *Dryas*, pick plot areas and set out pollination cages.

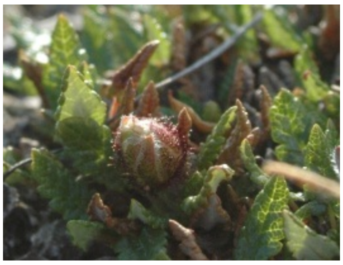

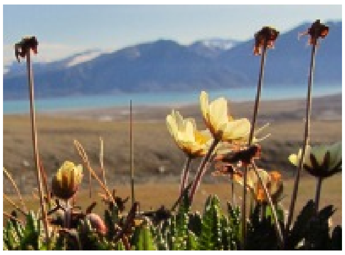

When *Dryas* buds are visible but still dark (pictured left), select five sampling plots of 1x1 m each. Individual plots should be 1-2 metres apart, randomly placed within *Dryas*-dominated vegetation and containing high densities of *Dryas* flowers. Look for the dried stalks of last year's flowers (pictured right). Please avoid highly disturbed sites like road verges – but do not select any overly inaccessible site, as you will need to keep an eye on it to detect when *Dryas* reaches peak flowering (step 2).

Start by marking one of the corners of the plot, then use a 1 m stick or 1x1 m frame (not supplied) to mark the other corners with small flags (supplied). Half the flags are colour coded: give each plot its own colour (two colour coded flags + two plain flags per plot), so we will know which flowers are from which plot.

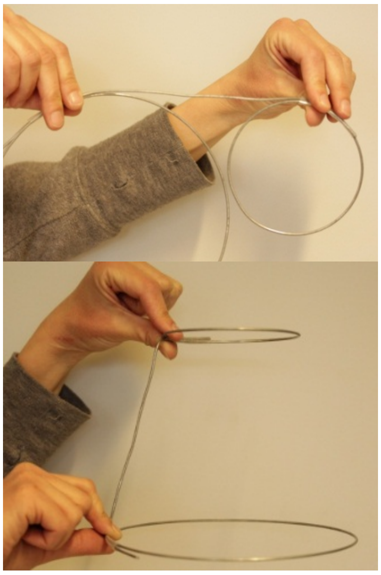

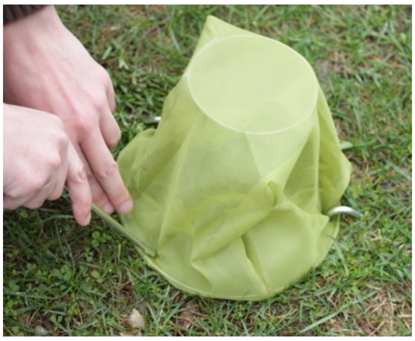

Within each plot, place two small cages (supplied) over *Dryas* cushions with plenty of buds. Bend the metal frames between the sections of clear tape, so that the rings are parallel with the small one on top (pictured left). Drape a cloth hood over the cage and fold the edges of the hood inwards under the bottom of the frame. Attach the cages firmly to the ground with three tent pegs pushed through both layers of the fabric fold (pictured right), while placing the two cages at least 10 cm apart.

Please take a picture of the site and its surroundings and fill in sampling form 1. Email the picture to sfegtweets@gmail.com.

The purpose of these cages is to exclude all pollinators while allowing the wind to pass through, thereby offering a baseline with which to gauge the contribution of insect pollinators.

Over the next few days, do keep an eye on the buds. Given good weather, *Dryas* will advance from dark buds to open flowers in less than a week. Once it seems that at least a third of the flowers are open, progress to STEP 2.

STEP 2. *About 2-3 hrs. Including half an hour of preparation at your base. Three days later, about an hour to collect and pack trap flowers. Today you will need the sticky flowers and yellow centers, the Dryas leaf envelope, sampling forms 2 and 3 and the extra flags.*

Once *Dryas* reaches maximum flowering (see above), return to the plots to record flower densities and phenology and to deploy trap flowers. Pick a sunny day if possible.

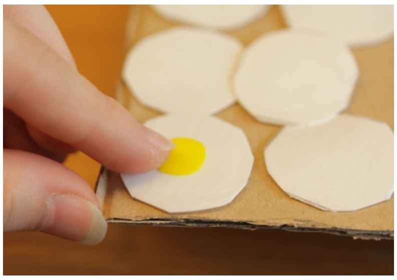

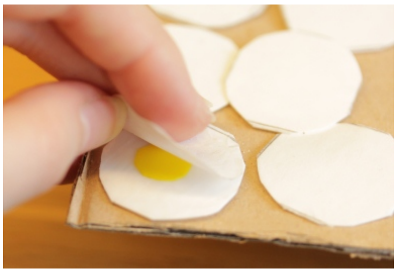

Before going out, assemble the trap flowers. This is best done indoors, as the pieces of cover paper can easily fly off and litter. It should also be done on the same day as the flowers are put out, as once the cover papers have been taken off the trap glue starts slowly drying. Remove and set aside the cover paper on the white flower base. Remove and throw away both cover papers on the yellow flower center. Tweezers can help here to avoid sticking them to your fingers. Place the yellow center in the middle of the flower base (pictured left) and cover again with the white flower's original cover paper (pictured right).

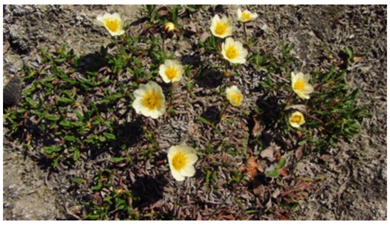

For each of your five plots, record the total number of flowers in each of the four categories on sampling forms 2 and 3 (outside and inside pollinator exclusion cages separately). If flower densities are high, you will find it hard to keep track of flowers already counted. In this case, please use additional sticks (supplied) to mark off smaller areas within each plot to first count one by one, then add up the counts. (Please remove these additional flags after counting.)

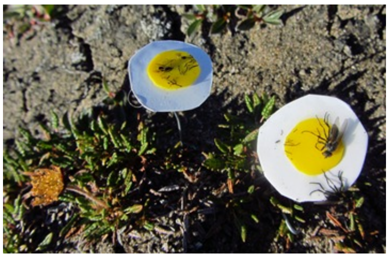

To deploy trap flowers, remove the protective cover and distribute twenty flowers evenly within each of your five sampling plots. Get the flowers out of the cardboard by straightening out the metal stem and pushing out from underneath. Do not pull on the sticky flower part or it may detach from its stem. Distribute the flowers into plots by the colour code on the cardboard base: the flowers in one plot should all have the same colour code as the plot flags. Stick the flowers firmly into the ground, until flower heads are about level with natural *Dryas* flowers (picture). Leave the trap flowers exposed for three days (ca 72 hours).

Collect small samples of *Dryas* leaves as directed on the separate envelope, either when putting out the sticky flowers or when collecting them three days later, whenever is more convenient.

After three days, collect the flowers, and pin them back into the cardboard base as they were before, bending the stems underneath. Use the colour codes to pin the flowers back into the same zones they were in before. Be careful not to touch the surface of the traps or the insects attached too much, as they are both truly sticky and fragile.

Place the envelope with the *Dryas* leaf samples and the filled-out forms on the bottom of the shipping box. On top of them, push down the cardboard with the sticky flowers. Store the box in a dry and preferentially cool place. As soon as possible, send to Helsinki according to separate instructions (supplied).

STEP 3 *About 2-3 hrs.* *Today you will need sampling forms 4 and 5, and something to carry the cages and flags away in.*

Late in the summer, when most of the *Dryas* flowers are done flowering and have either formed seed heads or dried (pictured right), return to the plots to record resultant seed set. For each of your five plots, record the total number of flowers in each of the four categories in sampling forms 4 and 5 (flowers outside and inside the pollinator exclusion cages separately). If the densities of seed heads are high, you will find it hard to keep track of heads already counted. In this case, please use the same flags as in STEP 2 (supplied) to mark off smaller areas within each plot to first count one by one, then add up the counts.

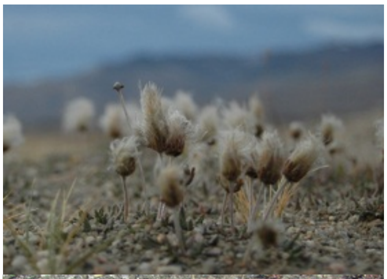

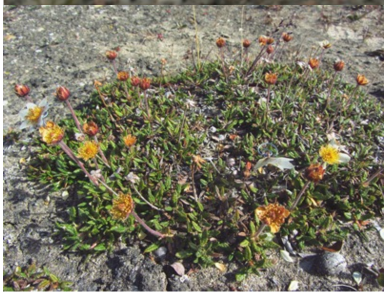

You may now remove and throw away all cages and flags. Please only return the sticky flowers, forms and leaf sample envelopes. Return sampling forms 4 and 5 by scanning and emailing to bess.hardwick@helsinki.fi, or by mailing with the included envelope.

THANK YOU FOR YOR CONTRIBUTION! Should you have the smallest question, please do not hesitate to contact us at bess.hardwick@helsinki.fi
